# Assessing the Sealing Performance and Clinical Outcomes of Endodontic Treatment in Patients with Chronic Apical Periodontitis Using Epoxy Resin and Calcium Salicylate Seals

**DOI:** 10.3390/medicina59061137

**Published:** 2023-06-13

**Authors:** Razvan Mihai Horhat, Bogdan Andrei Bumbu, Laura Orel, Oana Velea-Barta, Laura Cirligeriu, Gratiana Nicoleta Chicin, Marius Pricop, Mircea Rivis, Stefania Dinu, Delia Ioana Horhat, Felix Bratosin, Roxana Manuela Fericean, Rodica Anamaria Negrean, Luminita Maria Nica

**Affiliations:** 1 Department of Conservative Dentistry and Endodontics, Faculty of Dental Medicine, “Victor Babes” University of Medicine and Pharmacy Timisoara, Eftimie Murgu Square 2, 300041 Timisoara, Romania; horhat.razvan@umft.ro (R.M.H.); orel.laura@umft.ro (L.O.); velea.oana@umft.ro (O.V.-B.); cirligeriu.laura@umft.ro (L.C.); nica.luminita@umft.ro (L.M.N.); 2 Advanced and Digital Endodontic, Restorative and Prosthodontic Treatment (TADERP) Research Center, Department of Conservative Dentistry and Endodontics, Faculty of Dental Medicine, “Victor Babes” University of Medicine and Pharmacy Timisoara, Eftimie Murgu Square 2, 300041 Timisoara, Romania; 3 Department of Dental Medicine, Faculty of Medicine and Pharmacy, University of Oradea, 410073 Oradea, Romania; bogdanbumbu@uoradea.ro; 4 Faculty of General Medicine, “Vasile Goldis” Western University of Arad, Bulevardul Revolutiei 94, 310025 Arad, Romania; gchicin@gmail.com; 5 National Institute of Public Health, Strada Doctor Leonte Anastasievici 1-3, 050463 Bucharest, Romania; 6 Discipline of Oral and Maxillo-Facial Surgery, Faculty of Dental Medicine, “Victor Babes” University of Medicine and Pharmacy Timisoara, Eftimie Murgu Square 2, 300041 Timisoara, Romania; pricop.marius@umft.ro; 7 Department of Anesthesiology and Oral Surgery, Multidisciplinary Center for Research, Evaluation, Diagnosis and Therapies in Oral Medicine, “Victor Babes” University of Medicine and Pharmacy Timisoara, Eftimie Murgu Square 2, 300041 Timisoara, Romania; 8 Department of Pediatric Dentistry, Faculty of Dental Medicine, “Victor Babes” University of Medicine and Pharmacy Timisoara, Eftimie Murgu Square 2, 300041 Timisoara, Romania; stefania@dr-dinu.com; 9 ENT Department, “Victor Babes” University of Medicine and Pharmacy Timisoara, 2 Eftimie Murgu Square, 300041 Timisoara, Romania; horhat.ioana@umft.ro; 10 Department of Infectious Diseases, “Victor Babes” University of Medicine and Pharmacy, Eftimie Murgu Square 2, 300041 Timisoara, Romania; felix.bratosin@umft.ro (F.B.); manuela.fericean@umft.ro (R.M.F.); 11 Faculty of Medicine and Pharmacy, University of Oradea, 410073 Oradea, Romania

**Keywords:** endodontic treatment, apical periodontitis, endodontic sealer

## Abstract

*Background and Objectives:* Recognizing the significance of a hermetic apical seal for successful root canal treatment, the present investigation aimed to evaluate two sealing materials through an in vitro analysis, as well as to ascertain the clinical outcomes of patients treated with these two sealers in an in vivo setting. *Materials and Methods:* For the in vitro part of the study, two control groups of thirty monoradicular teeth were obturated with two sealers. Then, the sealers’ performance was tested based on a predefined protocol. Group A included 30 patients treated with an epoxy oligomer resin-based sealer (Adseal, MetaBiomed), while Group S comprised 30 patients treated with a polymeric calcium salicylate-based sealer (Sealapex, Kerr). Samples were sectioned and evaluated under the microscope to determine the sealer’s tightness by measuring the dye penetration into the root canal filling. For the in vivo part, a prospective study was designed to include 60 patients with chronic apical periodontitis in two endodontic treatment groups, using the same two sealers. *Results:* The in vitro analysis found that dye penetration in Group A was 0.82 mm (±0.428), while in Group S, the dye penetration was statistically significantly deeper, being 1.23 mm (±0.353). In the in vivo part of the study, the periapical index (PAI) significantly decreased 6 months after endodontic treatment, with 80.0% of patients in Group A having a PAI score of 2 compared to only 56.7% in Group S (*p*-value = 0.018). Similarly, tooth mobility scores significantly decreased after treatment, but with no difference between groups. The marginal bone loss decreased significantly more in the Adseal group compared to the Sealapex group (23.3% vs. 50.0%, *p*-value = 0.032). At the same time, 40.0% of patients in Group S had failed tooth healing compared to only 13.3% in Group A (*p*-value = 0.048). *Conclusions:* The in vitro study showed that Adseal had a better sealing capacity and a lower degree of dye penetration compared to Sealapex. However, on clinical evaluation in the in vivo study, both patient groups exhibited significant improvements in periapical index, tooth mobility scores, and pain reduction following endodontic treatment. Nevertheless, patients treated with Adseal showed a significantly greater improvement in PAI values, tooth mobility, and teeth healing after treatment. Overall, Adseal, as an endodontic sealer, may provide better sealing capabilities and enhanced clinical outcomes in the treatment of chronic apical periodontitis.

## 1. Introduction

The condition known as periodontitis is one of the most prevalent dental diseases that can lead to tooth loss [1]. Abnormalities in the periradicular bone that increase the risk of bacterial invasion and infection are often linked with this condition, which tends to spread to the gums, periodontal ligament, and the bone surrounding the teeth [2,3]. Apical periodontitis may include periapical abscess, acute apical periodontitis, chronic apical periodontitis, periapical granuloma, and radicular cyst [4,5]. In the field of contemporary endodontics, the management of apical periodontitis will continue to be of significant interest. According to the literature [6], the healing rate may reach up to 90% if managed accordingly on time, while the failure to heal can be linked to non-hermetic root canal filling performed during the endodontic treatment [7].

The ideal endodontic treatment involves two main objectives, including correct biomechanical cleaning, followed by the three-dimensional sealing of the endodontic system [8]. The efficient instrumentation reduces the number of bacteria in the root canal system and removes all the organic tissue and debris, therefore providing a higher longevity of the endodontic treatment [4,8]. Despite all the advances in shaping and disinfection techniques, studies have shown that the root canal space will still be colonized by bacteria [9]. If not sealed properly, the conjunction of remaining bacteria in the root canal system and leakage at the apex will have an unfavorable effect on the outcome and long-time success of the treatment [10,11].

Although gutta-percha remains the traditional commonly used material for core filler, other materials can be used as sealers between the dentin walls of the root and the core material [12]. Nevertheless, none of the available sealer agents can obtain an ideal perfect seal [13,14]. After calcium silicate cement was introduced, it demonstrated good results in terms of its sealing capacity and biocompatibility compared to other materials [15]. However, there are noted shortcomings with calcium silicate cement, particularly concerning the second-generation materials that take powder/liquid forms. One of the major drawbacks is their solubility, which can compromise the longevity and effectiveness of the seal [16]. For the third-generation materials that are ready-to-use, the setting time has been observed to be unpredictable, adding complexity to the clinical application [17]. Despite these limitations, calcium silicate cements, specifically calcium salicylate-based sealers, have been widely adopted in clinical practice due to their therapeutic properties. They have been designed to facilitate osteogenesis and cementogenesis while also providing antimicrobial protection. However, these sealers require use within a matrix for better sealing outcomes due to their solubility in water, which may result in instability over time [18,19].

In order to overcome these limitations, epoxy resin-based sealers, such as Adseal (MetaBiomed Co., Ltd, Chungbuk, Republic of South Korea) were engineered [20]. These sealers are composed of low-molecular-weight epoxy resins and amines, with a polymer being formed by an additional reaction between the epoxide groups and the amines [21]. They are considered resistant to moisture and solvents when cured, with low solubility and adherence to the root canal walls. To evaluate these properties in clinical practice, two studies investigated the postoperative pain as clinical outcome after root canal filling with traditional and new fillers [22,23]. It was described that the gutta-percha filling technique may positively affect the clinical outcomes compared with bioceramic materials, although the epoxy-resin sealer had better outcomes in terms of pulp status and number of visits.

Considering the properties of currently used sealers mentioned above and the hypothesis that Adseal is non-inferior to the Sealapex sealer, the main objective of the current study is to compare the sealing capacity and the apical micro-leakage of two classes of sealers: epoxy resin-based sealers (Adseal) versus salicylate-based sealers (Sealapex). As a secondary aim, the current study plans to observe and compare the clinical outcomes of the patients treated with these two different sealers.

## 2. Materials and Methods

### 2.1. Study Design

**Figure 1:**
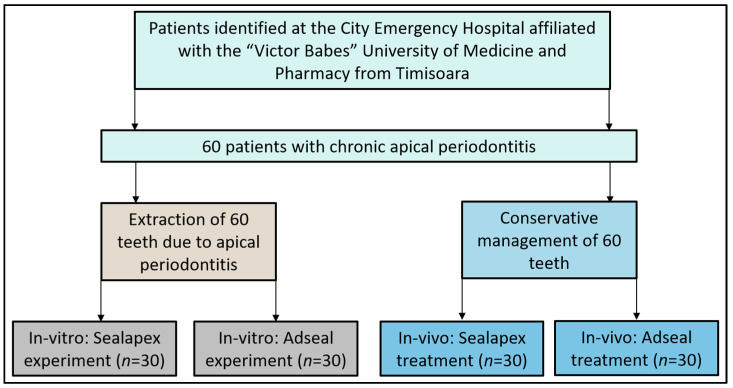
Flowchart of the study design. A total of 60 patients and 120 teeth were evaluated in the in vitro and the in vivo clinical study.

The current study followed a prospective cohort design, being conducted according to the guidelines of the Declaration of Helsinki and approved by the Ethics Committee of the City Emergency Hospital from Timisoara, affiliated with the “Victor Babes” University of Medicine and Pharmacy from Timisoara (approval number I-27098). All participants were individually informed of the study’s purpose and were requested to sign the consent form if they agreed to participate. Anonymization of patients’ personal data was further performed for privacy reasons. Sixty single-rooted teeth were extracted for the in vitro part of the study from 60 individual patients with a diagnosis of chronic apical periodontitis. Another 60 teeth from the same patients that required conservative management were included in the clinical part of the study. All patients were carefully selected after consenting to participate in this research. The diagnosis of chronic apical periodontitis was based on the International Classification of Diseases (ICD-10) [24], as seen in Figure 1. Chronic apical periodontitis was classified based on the severity and extent of the infection, as well as the presence or absence of symptoms, using the American Association of Endodontists (AAE) [25].

Inclusion criteria for the current study comprised the following: (1) patients older than 18 years; (2) patients’ informed consent for study participation; (3) the selection of monoradicular teeth with one straight root canal confirmed radiographically, with no prior restorative or endodontic treatment, that were affected by chronic apical periodontal disease; (4) patients who presented at the six-month follow-up.

The exclusion criteria set for this study were purposefully defined to ensure that our results were not biased by confounding variables or factors. Patients who were lost during the follow-up period were excluded to guarantee that the data sets were complete for all study participants, as incomplete data could result in misleading results and interpretations. Exclusion criteria comprised the following: patients lost at follow-up, tooth losses, patients with asymptomatic apical periodontitis, acute apical abscess, chronic apical abscess, condensing osteitis, irreversible pulpitis, necrotic pulp or previously treated pulp, teeth with cracks or fractures, root calcifications, root resorptions, large cavities, large carious lesions, generalized periodontitis, and root curvatures higher than 10°. These conditions, each representing a different stage or type of pulp and periapical diseases, could potentially have different responses to treatment, which might introduce confounding factors into our results.

### 2.2. In Vitro Study

After extraction, the teeth were numbered from 1 to 60 and randomly allocated to one of the two study groups, resulting in 30 teeth per group. Considering the sample size was relatively small, the random assignment was carried out using a random number generator. Group A was treated with Adseal, while Group S was treated with Sealapex. Obturation in both groups was performed using the Continuous Wave of Condensation (CWC) technique [26] with gutta-percha as the core material.

After filling the root canal system, all the samples were given two coats of clear nail varnish on the exterior root surface, leaving the last 1 mm apical portion uncoated. They were stored in an incubator for 72 h in a high-humidity environment and at a constant 37 °C temperature. The samples were kept in a 2% methylene blue dye for one week and left to dry for 24 h at room temperature after rinsing them under running water. Afterward, the teeth were vertically split using a separating disc by the same operator.

All the samples were observed under a dental operating microscope (COXO, Foshan Coxo Medical Instruments Co., Ltd, Foshan, China) with a magnification of 10× to measure the dye penetration at a millimetric scale, then measured using a hand caliper with a precision of 0.1 mm. Measurements included the total working length (WL) in millimeters and the degree of percolation in millimeters measured from the apical foramen (i). A scoring system for the penetration (i) was also used (score 0 for i < 1 mm and score 1 for i ≥ 1 mm). Two measurements were made according to the two halves of each sample tooth. For the samples in which different values were recorded, the highest one was chosen to be reported.

### 2.3. Clinical (In Vivo) Study Protocol

The same patients with chronic apical periodontitis that had one tooth extracted for the in vitro part of the study also participated in the clinical study where another tooth from each patient required conservative management. Two retro-alveolar X-rays were taken for each patient, one after root canal shaping and one after obturation. One practitioner (R.M.H.) with ten years’ experience in endodontics performed all endodontic treatments under a dental microscope. The access cavity for all the teeth was performed minimally invasive, with a diamond round long-necked burr, after which the working length was determined using an ISO 10 K-file (Kendo, VDW GmbH, Munich, Germany) and an endodontic ruler. The location of the rubber stopper was maintained on the coronal surface during the entire shaping procedure.

The canal negotiation phase followed, using #0.10 and #0.15 K-files (Kendo, VDW), after which the teeth samples were shaped using the ProTaper Next System, using an X1 0.17 mm diameter file, 4% taper (17/0.04), and a primary X2 with a 0.25 mm tip diameter and 6% taper (25/0.06) in conjunction with the X-Smart endodontic motor (Dentsply Maillefer, Ballaigues, Switzerland). According to the torque chart of the manufacturer, it was used at 300 rpm. The root canal systems were irrigated during shaping with NaOCl 5.25% solution 1:1 (Cerkamed, Stalowa Wola, Polska).

The obturation technique used for all the teeth was the Continuous Wave of Condensation, with the System B-Fill and Pack (SybronEndo, Orange, CA, USA). Using adapted ProTaper Next X2 gutta-percha points and sealer, thermoplasticized gutta-percha was injected for back-fill. The cleaned and shaped teeth were divided into groups, Group S and Group A, each assessing an equal number of teeth (*n* = 30). Group S teeth samples were sealed using calcium salicylate agents (Sealapex, Kerr, Orange, CA, USA). In contrast, Group A samples were sealed using epoxy-resin agents (Adseal, MetaBiomed Co., Ltd., Chungbuk, Republic of Korea). The same operator performed all the endodontic treatments to prevent biased results.

### 2.4. Data Collection and Study Variables

After all the measurements were taken, data were recorded in Microsoft Excel on a Pro-forma template. Patients’ characteristics considered relevant for analysis in the current study comprised: (1) demographic and background characteristics—age, gender, body mass index, number of comorbidities, diabetes mellitus, smoking history, teeth brushing habits; (2) dental characteristics—position of the studied tooth, the proportion of patients with mobile teeth and missing teeth, periodontitis stage, periodontitis grade, the extent of periodontitis, ride defect; and (3) patients’ evolution before and after treatment using the periapical index (PAI) [27], tooth mobility score, periodontal pocket depth, presence of pain at the site of intervention, sealer extrusion, marginal bone loss, and tooth healing. The distribution of the PAI index was calculated after assigning the two study groups to provide a uniform distribution before performing the experiment.

### 2.5. Statistical Analysis and Measurements

The sample size was determined using a convenience sampling method due to its feasibility and efficiency for the study’s circumstances. It was calculated that 97 cases represent the ideal sample size for a margin of error of 10% and a confidence level of 95%. The threshold for statistical significance was 0.05. The statistical power (1-β) calculation was 80% for a type I error rate of 5%. For the statistical analysis, normal distribution of the continuous data was assessed with the Kolmogorov–Smirnov test, and we calculated the mean and the standard deviation (SD) for all Gaussian variables. Student’s *t*-test with two-factor comparisons was used to compare the normally distributed data. Fisher’s exact test was performed to compare the proportions of penetration scores and categorical data. All *p* values < 0.05 were accepted as statistically significant. All data were processed using Statistical Package for the Social Sciences (SPSS) v.26 for Windows (IBM, Armonk, NY, USA).

## 3. Results

### 3.1. Experimental (In Vitro) Study

**Figure 2:**
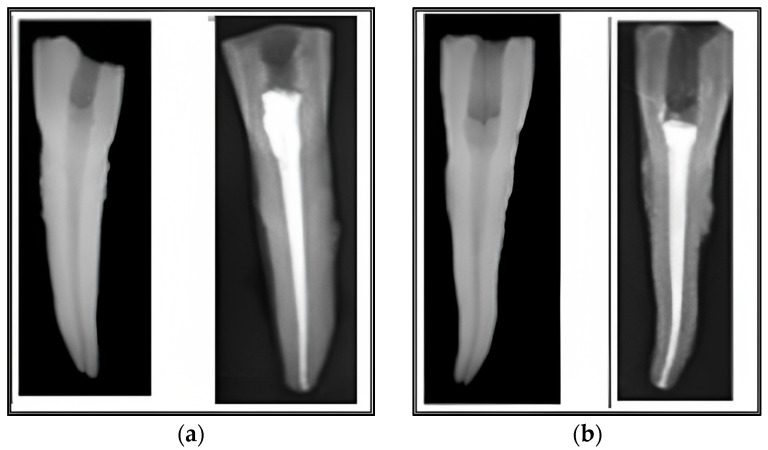
Periapical X-ray of one sample from each group after shaping (left) and at the end of the root canal obturation (right): (**a**) Adseal; (**b**) Sealapex.

**Figure 3:**
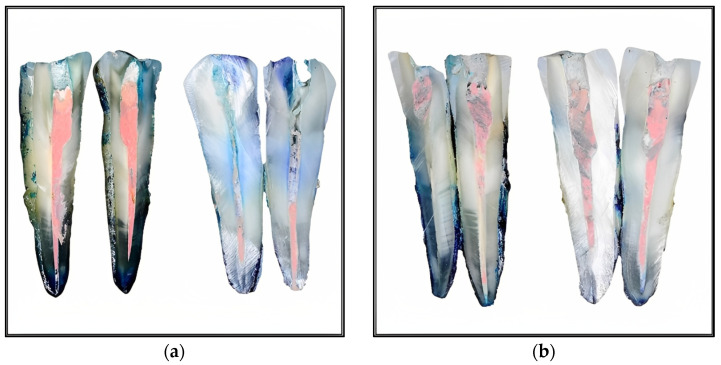
Images of two longitudinally sectioned samples from each group, showing the penetration of the methylene blue dye along the apical part of the root canal filling: (**a**) Adseal; (**b**) Sealapex.

At the radiological evaluation after root canal filling, no differences were observed between the two groups regarding the quality of the performed treatments. The root canal filling was regarded as appropriate if it was of sufficient length, contained no voids, and tapered consistently from the aperture to the apex. All teeth were obturated on full working length, with a good seal at the foramen and along the root canal walls, with no radiographic distinction between the two sealers nor between the used sealer and the thermoplasticized gutta-percha for each group (Figure 2a,b). The vertically split teeth were analyzed under the dental operating microscope, and pictures of each half were taken using a Canon EOS 30D camera (Figure 3a,b).

### 3.2. Sample Measurements

The mean values of the working length (WL), length of penetration (i) measured in mm from the apical foramen, the ratio between the percolation and the working length (i/WL), the standard deviation, and the penetration scoring for each group were recorded in Table 1 and measured from the same set of teeth. The mean (±SD) WL in Group A was 19.22 mm (±1.1194), while the mean (±SD) WL in Group S was 19.32 mm (±1.1179). When performing the Student’s two-factor *t*-test, we calculated (*p*-value = 0.730), meaning that no significant statistical differences regarding the working length measurements of the root canals between groups were recorded, thus confirming the similarity of the two groups.

### 3.3. Analysis of the Degree of Penetration

**Figure 4:**
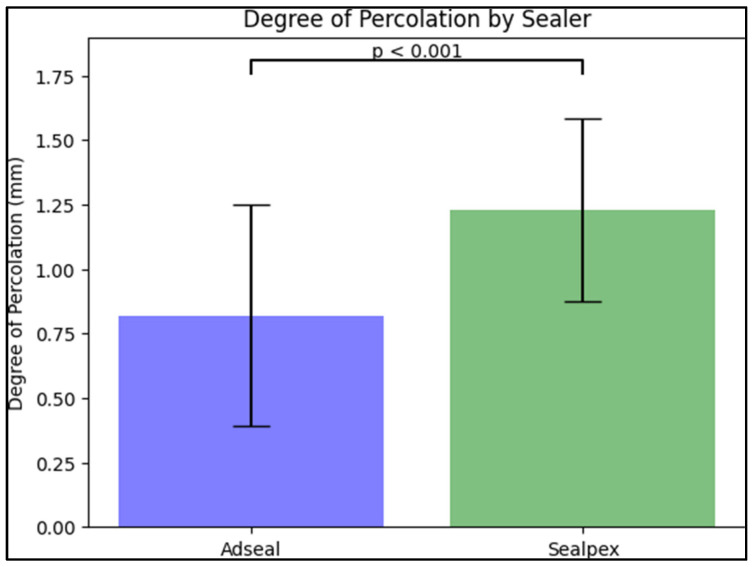
Comparison of the degree of percolation measured in millimeters between patients treated with Adseal and Sealpex.

The mean of “i” in Group A was 0.82 mm (±0.428), while the mean “i” in Group S was 1.23 mm (±0.353) (Figure 4). When performing a Student’s two-factor *t*-test, we calculated *p* < 0.001 and *p* < 0.05, resulting in the rejection of the null hypothesis that there are no significant statistical differences regarding the penetration measurements between the two groups. Thus, it was confirmed that in Group A, sealed with Adseal, a lower degree of dye penetration was obtained than in Group S, sealed with Sealapex.

The scoring for penetration was compared using Fisher’s exact test by analyzing a contingency table of 2 × 2 (Table 1), resulting in a statistically significant difference (*p*-value = 0.003). As such, teeth treated with Adseal are more prone to receive Score 0 (with less than 1 mm of dye penetration), and teeth treated with Sealapex are more prone to receive Score 1 (with greater or equal to 1 mm of dye penetration). Therefore, we can affirm that Group A has better values when we look into the ability to seal the root canal system, as we can observe less of the working length being exposed to external fluids. As such, Adseal has a better sealing capacity within the endodontic space.

### 3.4. Patients’ Characteristics

The general characteristics of the sixty patients with chronic apical periodontitis among the two study groups presented in Table 2 did not identify any significant differences. The study participants were matched by age, number of comorbidities, and smoking status. The mean age in Group A was 52.6 years and 53.4 years in Group S, while the majority were men (60.0% in Group A vs. 56.7% in Group S). The number of comorbid conditions did not differ significantly between the study groups, but it was observed that more than 30% of all participants had diabetes mellitus. Teeth brushing was inconsistent with at least one daily brushing, as 46.7% of patients in Group A admitted brushing less than once a day; in Group S, 43.3% brushed less than once a day.

### 3.5. Patients’ Outcomes

Table 3 illustrates the dental characteristics of the two study groups, Group A (*n* = 30) and Group S (*n* = 30). When comparing the position of the studied teeth, there was no significant difference between the two groups (*p* = 0.808). In Group A, most of the teeth that were treated were mandibular premolars, in a proportion of 30.0%, while in Group S, 36.7% were mandibular premolars. Regarding other teeth characteristics, there was no significant difference in the proportions of mobile teeth (*p* = 0.573) and missing teeth (*p* = 0.726) between the two study groups. Group A had 73.3% mobile teeth and 30% missing teeth, while Group S had 66.7% mobile teeth and 40% missing teeth.

The periapical index (PAI) distribution also showed no significant difference between the groups (*p* = 0.590). In Group A, the majority of patients had a PAI of 3, and only 16.7% had a PAI of 4. In Group S, the distribution was 56.7% of patients with PAI 3 and 26.7% with PAI 4. For periapical bone radiolucency, no significant difference was observed between the groups (*p* = 0.590). In Group A, the majority of samples had a radiolucency of 2–4 mm (63.3%), and 20% had a radiolucency greater than 4 mm, while in Group S, the highest proportion was 73.3% for a radiolucency of 2–4 mm. Lastly, the distribution of ridge defects between the groups did not reveal any significant difference (*p* = 0.405). In Group A, 63.3% had mild ridge defects (<33%), and 36.7% had moderate ridge defects (33–50%). In Group S, these percentages were 73.3% and 26.7%, respectively.

The patient follow-ups described in Table 4 identified multiple significant differences after endodontic treatment before and 6 months after treatment, as well as between the two study groups stratified by sealer composition. It was observed that both groups demonstrated a statistically significant improvement in PAI scores following the treatment. In Group A, the proportion of patients with a PAI of 2 increased from 23.3% before treatment to 80% after treatment, while in Group S, the proportion increased from 16.7% to 56.7% (*p* = 0.018, Cohen’s h = 0.423).

Regarding other characteristics, tooth mobility scores equal or greater than two significantly decreased after treatment in both groups, with no significant difference between the groups (*p* = 0.316, Cohen’s h = 0.696). The presence of pain at the site of intervention significantly reduced after treatment in both groups (*p* < 0.001 for Group A and *p* = 0.002 for Group S), with no significant difference between the groups (*p* = 0.416. Cohen’s h = 0.502). Sealer extrusion was observed in 6.7% of Group A patients and 16.7% of Group S patients, with no significant difference between the groups (*p* = 0.227, Cohen’s h = 0.522). However, a significant difference in marginal bone loss was observed between the groups (*p* = 0.032, Cohen’s h = 0.485), as well as regarding tooth healing (*p* = 0.048).

## 4. Discussion

### 4.1. Analysis of Results and Literature Findings

Overall, the current study identified potential benefits of Adseal over Sealapex in patients treated for chronic apical periodontitis. The main in vitro experiment findings of this prospective study showed that dye penetration in Group S treated with Sealapex was statistically significantly deeper than in Group A treated with Adseal. Clinical outcomes such as PAI, bone loss, and tooth healing were also significantly improved at the six-month follow-up.

Dye penetration in our study was measured to indirectly determine the fluid-proof sealing of the materials used. To achieve a fluid-proof seal of the root canal system, various types of endodontic sealers are used in association with a core material in the obturation step of the endodontic treatment [28,29]. As the literature shows, an ideal root canal sealer should have an excellent seal, dimensional stability, a long enough setting time to allow the root canal system’s obturation, insolubility against tissular fluids, proper canal wall adhesion, and biocompatibility [30,31,32,33].

Similarly to what was observed in the current research, higher levels of dye penetration in the Sealapex group can be explained by its high solubility and low stability compared to epoxy-resin-based sealers [34,35,36,37,38,39,40,41]. In a comparative study, epoxy resin-based sealers exhibited better dimensional stability than calcium salicylate-based sealers, even after immersion in water and artificial saliva for up to 28 days [42,43,44]. Additionally, two studies showed that after being stored in water for a longer period (between 14 and 28 days), Sealapex demonstrated a strong sealing ability initially but poor sealing capacity afterward, which could be due to sealer disintegration [39,45]. However, in the study of Schafer et al. [46], Sealapex presented a higher degree of weight loss in comparison with other classes of endodontic materials after immersion in distilled water or artificial saliva for variable periods (8 h to 28 days), which may also explain the higher degree of dye penetration observed in the present study for Sealapex samples.

Another important and highly desired characteristic of a sealer is represented by its setting time, as it can affect the quality of a hermetic root canal seal [45,46,47,48]. Contradictory results regarding the sealing ability of calcium salicylate-based sealers have been reported in the literature due to differences in research protocols [37]. Some studies have shown better sealing properties for calcium salicylate-based sealers four weeks after setting, while epoxy resin-based sealers showed better results after seven days [32]. In our study, we considered a 72 h setting time before the seven-day sample immersion in methylene blue dye solution to evaluate the linear penetration and sealing ability of the tested sealers, which is consistent with previous research [49,50]. Therefore, dye penetration studies should be performed after the complete set of sealers. In the present study, samples were left to set for 72 h after root canal filling in both groups because contradictory data were found in the literature regarding the setting time of the two tested sealers.

For Adseal, the existing literature describe a setting time of 70 ± 9 min, which is ten times shorter than that of AH Plus (712 ± 95 min) [40], but other studies reported that the final setting time of epoxy resin-based sealers might vary by about six hours [38]. For Sealapex, an initial setting time of 58 min and a good initial sealing ability were reported, meeting ISO 6876:2012 standard requirements [51], which recommends a setting time between 30 min to 72 h for endodontic sealers [24]. Another study showed a setting time of 912 ± 14 min for Sealapex [50], but with time, because of its volumetric expansion in high-humidity environments, the sealing ability may be lost through the sealer’s dissolution and eventually lost over a variable period [30]. Orstavik et al. [50] reported in one study that Sealapex was the only sealer that could not be tested by any means because it did not set in humid environments. In addition, Andrade et al. [52] reported a final setting time for epoxy resin-based sealers of 28 days after the initial handling of these materials.

Three-dimensional sealing of the root canal system is the main objective in preventing apical micro-leakage [43]. It has been proven that epoxy-resin-based sealers can also adhere to gutta-percha and dentin, thus representing an advantage of this category of sealers [51]. Complete root canal filling using a biocompatible and dimensionally stable material is essential in achieving successful endodontic treatment [50]. At the same time, it has been observed and unanimously accepted that a complete hermetic seal of the three-dimensional endodontic space is impossible to achieve with the current materials and using current techniques; therefore, further clinical or in vitro studies should continue to assess the seal ability of endodontic materials [53,54]. As demonstrated, micro-leakage in the root canal filling is considered the single most important risk factor responsible for apical periodontitis [55].

Regarding the clinical outcomes observed in the current study, no significant differences were identified between the two groups regarding general patient characteristics, suggesting that any disparities in treatment outcomes would be attributable to the differences in the sealers themselves and not other confounding factors. Clinical findings such as the distributions of the periapical index (PAI) and ridge defects were also similar between the two groups, reflecting the comparability of the clinical manifestations of apical periodontitis in these patients. Consistent with prior studies, we observed a substantial reduction in PAI scores following endodontic treatment in both groups [56,57]. In line with other investigations, tooth mobility and pain at the intervention site were significantly reduced after treatment across both sealers [58,59].

Interestingly, our results suggest that treatment with Adseal may offer some advantages. Although sealer extrusion was not significantly different between the two groups, there was a lower rate of sealer extrusion in the Adseal group, an observation that warrants further study. Moreover, a significant difference in marginal bone loss was observed between the groups, favoring the Adseal sealer. This finding is intriguing, as it suggests a potential differential effect on bone preservation, which has not been widely reported in previous studies and therefore represents a novel contribution to the literature. Moreover, tooth healing also showed a significant difference between the groups, which might be attributed to the specific properties of the sealers used, and which may be further investigated in future studies.

Despite the difference between the sealing capabilities of the materials used for endodontic treatment, our study supports the notion that treatment in chronic apical periodontitis and periodontal disease is beneficial [60]. A deliberate endodontic intervention using combination therapy can be a beneficial strategy for the complex care of teeth with severe attachment loss, alveolar bone degeneration, and simultaneous secondary endodontic involvement.

Despite the hypothesis that Adseal is non-inferior to the Sealapex sealer, in future studies regarding the sealing ability of endodontic materials, a longer setting time for both sealers should be considered before testing, as more than 72 h might be required, thus being one of the limitations of the present study. Because of the limited number of studies found in the current literature regarding comparing Adseal and Sealapex sealers, further research studies are needed for a better overview of the penetration rate.

### 4.2. Study Limitations

A modest sample size of 60 patients and 60 teeth were used in the current investigation. The results may have been more clearly defined with additional research samples and more calibrated measurements of the root canal lengths, curvatures, and dye penetration. Because the current study was conducted over 6 months, future studies might be undertaken over a longer period to obtain more accurate results regarding the stability of the root canal system’s three-dimensional filling and its tight seal over time. Moreover, one of the potential limitations of this study pertains to the use of convenience sampling for participant recruitment. While this method provided practical benefits for this study in terms of resource allocation, accessibility, and time management, it inherently risks the potential for selection bias. Consequently, the study population may not be fully representative of the broader population of individuals with chronic apical periodontitis undergoing endodontic treatment. Another limitation to acknowledge involves the lack of operator blinding during the endodontic treatments. This omission could potentially introduce an element of operator bias, where knowledge of the type of sealer being used may influence the operator’s technique or decision-making process during the procedure. Moreover, the outcomes of endodontic treatments can be significantly influenced by the skill and experience of the operator.

## 5. Conclusions

In conclusion, our study demonstrated that the sealing efficiency and clinical outcomes in patients with chronic apical periodontitis undergoing endodontic treatment varied between epoxy-resin and calcium salicylate sealers. The Adseal (epoxy-resin) group showed better sealing capacity and a lower degree of dye penetration (0.83 mm) compared to the Sealapex (calcium salicylate) group (1.23 mm). However, both groups exhibited significant improvements in periapical index, tooth mobility scores, and pain reduction following endodontic treatment. Although there was no significant difference between the groups in terms of tooth mobility scores, periodontal pocket depth, pain presence, and sealer extrusion, the Adseal group showed a significantly greater improvement in periapical index values after treatment. Although significant clinical differences were not observed in all parameters, there might still be a clinically meaningful difference that our study was unable to detect due to sample size or other factors. Overall, this study suggests that the use of Adseal as an endodontic sealer may provide better sealing capabilities and enhanced clinical outcomes in the treatment of chronic apical periodontitis, but studies with a larger sample size can be useful to strengthen these findings.

## Figures and Tables

**Table 1:** Measurements and scorings of penetration for Group A and Group S.

Group	Measurement	WL (mm) *	i (mm)	i/WL (%)	Score 0	Score 1
Adseal (*n* = 30)	Mean (SD)	19.22 (1.11)	0.82 (0.42)	4.25% (2.16%)	18	12
Sealapex (*n* = 30)	Mean (SD)	19.32 (1.11)	1.23 (0.35)	6.38% (1.80%)	6	24

WL—Working length; A—Adseal; S—Sealapex; Score 0—percolation <1 mm; Score 1—percolation ≥1 mm; SD—Standard deviation; *—Data analyzed using Student’s *t*-test (*p*-value = 0.730).

**Table 2:** General characteristics of the two study groups.

Variables *	Group A (*n* = 30)	Group S (*n* = 30)	*p*-Value
General characteristics			
Age (years), mean (SD)	52.6 (4.6)	53.4 (5.2)	0.530
Gender (men)	18 (60.0%)	17 (56.7%)	0.793
BMI, mean (SD)	23.7 (2.8)	23.3 (3.6)	0.632
Comorbidities			0.710
0–1	14 (46.7%)	17 (56.7%)	
2	9 (30.0%)	8 (26.7%)	
≥3	7 (23.3%)	5 (16.7%)	
Diabetes Mellitus	11 (36.7%)	10 (33.3%)	0.786
Smoking			0.602
Yes	18 (60.0%)	16 (53.3%)	
No	12 (40.0%)	14 (46.7%)	
Teeth brushing			0.777
Less than 1/day	14 (46.7%)	13 (43.3%)	
1/day	14 (46.7%)	16 (53.3%)	
More than 1/day	2 (6.7%)	1 (3.3%)	

* Data are presented as *n* (%) unless specified differently; BMI—body mass index; SD—standard deviation.

**Table 3:** Dental characteristics of the two study groups.

Variables *	Group A (*n* = 30)	Group S (*n* = 30)	*p*-Value
Position of the studied tooth			0.808
Maxillary molar	6 (20.0%)	7 (23.3%)	
Maxillary premolar	8 (26.7%)	5 (16.7%)	
Mandibular molar	7 (23.3%)	7 (23.3%)	
Mandibular premolar	9 (30.0%)	11 (36.7%)	
Other teeth characteristics			
Mobile teeth	22 (73.3%)	20 (66.7%)	0.573
Missing teeth	9 (30.0%)	12 (40.0%)	0.726
PAI			0.590
2	7 (23.3%)	5 (16.7%)	
3	18 (60.0%)	17 (56.7%)	
4	5 (16.7%)	8 (26.7%)	
Periapical bone radiolucency			0.590
1–2 mm	5 (16.7%)	4 (13.3%)	
2–4 mm	19 (63.3%)	22 (73.3%)	
>4 mm	6 (20.0%)	4 (13.3%)	
Ride defect			0.405
Mild (<33%)	19 (63.3%)	22 (73.3%)	
Moderate (33–50%)	11 (36.7%)	8 (26.7%)	

* Data are presented as *n* (%) unless specified differently; PAI—Periapical Index.

**Table 4:** Patients’ evolution before and after treatment.

Variables	Group A (*n* = 30)		*p*-Value	Group S (*n* = 30)		*p*-Value	Between Groups *
	Before	Follow-Up		Before	Follow-Up		
PAI			<0.001			<0.001	0.018
2	7 (23.3%)	24 (80.0%)		5 (16.7%)	17 (56.7%)		
3	18 (60.0%)	4 (13.3%)		17 (56.7%)	13 (43.3%)		
4	5 (16.7%)	2 (6.7%)		8 (26.7%)	0 (0.0%)		
Other characteristics							
Tooth mobility score ≥2	19 (63.3%)	4 (13.3%)	<0.001	17 (56.7%)	7 (23.3%)	0.008	0.316
Periodontal pocket depth ≥6 mm	16 (53.3%)	11 (36.7%)	0.194	13 (43.3%)	7 (23.3%)	0.100	0.259
Presence of pain at the site of intervention	26 (86.7%)	9 (30.0%)	<0.001	24 (80.0%)	12 (40.0%)	0.002	0.416
Other pain	22 (73.3%)	10 (33.3%)	0.002	23 (76.7%)	15 (50.0%)	0.032	0.190
Sealer extrusion	-	2 (6.7%)	-	-	5 (16.7%)	-	0.227
Marginal bone loss	14 (46.7%)	7 (23.3%)	0.058	20 (66.7%)	15 (50.0%)	0.190	0.032
Tooth healing							0.048
Healed	-	7 (23.3%)	-	-	3 (10.0%)	-	
Healing	-	19 (63.3%)	-	-	15 (50.0%)	-	
Failed	-	4 (13.3%)	-	-	12 (40.0%)	-	

Data analyzed using Chi-square test; * *p*-value comparing Group A vs. Group S; PAI—periapical index; Healed—Normal clinical status associated with PAI scores of 1 or 2; Healing—Other than pressure sensitivity, clinical normality is accompanied by a reduction in the extent of the periradicular lesion or a decrease in the PAI score; Failed—The occurrence of clinical signs and symptoms accompanied with a radiographic PAI score of 3 or higher, a rise in the extent of the periradicular lesions, or an increase in the PAI score was regarded to indicate a failed tooth procedure.

## Data Availability

The data presented in this study are available on request from the corresponding author.
